# Combined Atlas and Convolutional Neural Network-Based Segmentation of the Hippocampus from MRI According to the ADNI Harmonized Protocol

**DOI:** 10.3390/s21072427

**Published:** 2021-04-01

**Authors:** Samaneh Nobakht, Morgan Schaeffer, Nils D. Forkert, Sean Nestor, Sandra E. Black, Philip Barber, the Alzheimer’s Disease Neuroimaging Initiative

**Affiliations:** 1 Medical Sciences Graduate Program, University of Calgary, Calgary, AB T2N 1N4, Canada; 2 Department of Clinical Neurosciences, University of Calgary, Calgary, AB T2N 1N4, Canada; morgan.schaeffer@ucalgary.ca (M.S.); nils.forkert@ucalgary.ca (N.D.F.); pabarber@ucalgary.ca (P.B.); 3 Department of Radiology, University of Calgary, Calgary, AB T2N 1N4, Canada; 4 Department of Psychiatry, University of Toronto, Toronto, ON M5S, Canada; sean.nestor@mail.utoronto.ca (S.N.); sandra.black@sunnybrook.ca (S.E.B.); 5 Department of Medicine, Sunnybrook Health Sciences Centre, Toronto, ON M4N 3M5, Canada

**Keywords:** magnetic resonance imaging, hippocampus, segmentation, convolutional neural network, ADNI harmonized hippocampal protocol

## Abstract

Hippocampus atrophy is an early structural feature that can be measured from magnetic resonance imaging (MRI) to improve the diagnosis of neurological diseases. An accurate and robust standardized hippocampus segmentation method is required for reliable atrophy assessment. The aim of this work was to develop and evaluate an automatic segmentation tool (DeepHarp) for hippocampus delineation according to the ADNI harmonized hippocampal protocol (HarP). DeepHarp utilizes a two-step process. First, the approximate location of the hippocampus is identified in T1-weighted MRI datasets using an atlas-based approach, which is used to crop the images to a region-of-interest (ROI) containing the hippocampus. In the second step, a convolutional neural network trained using datasets with corresponding manual hippocampus annotations is used to segment the hippocampus from the cropped ROI. The proposed method was developed and validated using 107 datasets with manually segmented hippocampi according to the ADNI-HarP standard as well as 114 multi-center datasets of patients with Alzheimer’s disease, mild cognitive impairment, cerebrovascular disease, and healthy controls. Twenty-three independent datasets manually segmented according to the ADNI-HarP protocol were used for testing to assess the accuracy, while an independent test-retest dataset was used to assess precision. The proposed DeepHarp method achieved a mean Dice similarity score of 0.88, which was significantly better than four other established hippocampus segmentation methods used for comparison. At the same time, the proposed method also achieved a high test-retest precision (mean Dice score: 0.95). In conclusion, DeepHarp can automatically segment the hippocampus from T1-weighted MRI datasets according to the ADNI-HarP protocol with high accuracy and robustness, which can aid atrophy measurements in a variety of pathologies.

## 1. Introduction

Dementia has devastating effects on patients as well as their families and care givers [1]. It is now becoming apparent that for new treatments to be effective, patients will have to receive preventative or disease modifying drugs in the earliest, and possibly even pre-symptomatic stages [2]. Pathological global and regional cerebral atrophy reflects neuronal cell loss that can be measured from serially acquired MRI scans even before the onset of cognitive impairment has been identified. For this reason, it has been suggested as a potential early identifiable precursor of cognitive decline [3]. Within this context, longitudinal hippocampus volume changes (atrophy rates) have been found especially valuable for the identification of subjects at risk of imminent cognitive decline [4,5]. Hippocampus atrophy can also be used to track disease progression and has the potential to serve as a study outcome measure in pre-dementia enrichment clinical trials [6,7]. Hippocampus atrophy has been found to be a reliable biomarker to identify subjects in the early stages of Alzheimer’s disease (AD) and to predict the time to progression to AD in subjects with mild cognitive impairment (MCI) who have amyloid positive results [8,9]. Cross-sectional population-based studies have also shown that the hippocampus volume and hippocampus atrophy rate can predict future cognitive decline in cognitively normal subjects 5–10 years before symptoms develop [8,9,10].

Accurate hippocampus volume measurement requires a robust and accurate segmentation tool. A growing body of research exists aiming to improve the accuracy of hippocampus segmentation for early diagnosis of dementia [11,12,13]. Hippocampus segmentation methods that incorporate population knowledge such as multi-atlas and machine learning techniques perform better compared to other established methods [12,14,15]. However, the accuracy of multi-atlas segmentation techniques is known to be affected by multiple factors such as the similarity of the atlas to the dataset to be segmented, the number of atlases in the library, the inter-operator variability of the manual segmentation atlas library, and the label fusion and registration algorithms used for segmentation. Moreover, high computational power is required for time efficient processing [12].

Segmentation methods that use machine learning algorithms, such as support vector machines, require many high-quality labelled datasets and a priori feature extraction [16]. Novel deep learning models have been shown to provide more generalizable results compared to traditional machine learning techniques for many segmentation tasks, but also require large training sets for optimal results [13,14]. Neuroanatomical segmentation using deep learning models has achieved significantly higher accuracies and reduced processing time compared to conventional segmentation algorithms used in the FSL [17] and Freesrufer algorithms [13,14,18]. For instance, QuickNat [14] used automatically generated segmentations of a large number of datasets for pre-training of a convolutional neural network (CNN) and a small number of manually segmented datasets for fine tuning, demonstrating a high level of accuracy. Another recently described technique, HippoDeep [13], used a large dataset of segmented hippocampi (>20 K) extracted by FreeSurfer [19] for pre-training of the CNN within a predefined bounding box containing both hippocampi, and fine-tuned parameters with a single manually labelled brain. Similar methodologies have been adopted by other groups [20]. Generally, most previously described deep learning methods for hippocampus segmentation include a pre-training step using large datasets and fine tuning of the CNN model using a small set of manually segmented datasets [13,14].

Even though CNN-based hippocampus segmentation methods yield better results compared to traditional segmentation methods, they are often biased by the quality of the ground truth hippocampus labels used for training the CNN. Recently, the Alzheimer’s Disease Neuroimaging Initiative (ADNI) defined the harmonized hippocampal protocol (HarP) for the manual segmentation of the hippocampus from structural MRI, which aims to reduce inter-observer differences [21,22]. ADNI-HarP is an international effort aiming to harmonize the hippocampus segmentation by defining a standard protocol that results in hippocampus segmentations that can be compared between different sites and clinical trials. ADNI made 135 T1-weighted MRI images publicly available that were manually segmented in the anterior commissure-posterior commissure plane following the ADNI-HarP guidelines. The protocol has high inter- and intra-rater agreement compared to other protocols not following the ADNI-HarP segmentation guidelines by clearly specifying anatomical landmarks for precise hippocampus segmentation [23].

According to protocol, the cut line between the amygdala and hippocampus at the rostral level can be recognized by segmenting gray matter below the alveus and at the caudal level by separating the last grey matter mass inferomedial to the lateral ventricles. The protocol suggests using 2D orthogonal views (axial, sagittal, and coronal planes) for optimal tracings. This recommendation relates to the similarity of tissue intensity of the surrounding tissues, in which case visualization in the different orthogonal views can help to assess the continuity of the structures, aiding segmentation, especially at the medial boundary of the hippocampus, where the grey matter is difficult to delineate based on only voxel intensity information. The ADNI-HarP protocol was agreed upon by a panel of experts from North America and Europe and is accepted as the standard systematic method for hippocampal manual segmentation [21,22].

The ground truth data include the brain MRI datasets and the corresponding manual hippocampus segmentations by ADNI-Harp experts. Their segmentations have more than 90% agreement [22].

To date, the majority of hippocampal segmentation tools developed either utilize atlas-based segmentation methods or deep learning-based algorithms, in particular CNNs [24,25,26,27], but given the novelty of ADNI-HarP do not produce segmentations following these guidelines. Therefore, the aim of this study was to develop a highly accurate atlas- and CNN-based hippocampus segmentation tool for MRI data, called DeepHarp, that generates ADNI-HarP standardized hippocampus segmentations. The CNN model was partially trained, fully validated, and tested using this data to ensure the competency of the model with expert manual tracing criteria developed by the ADNI Collaborators.

Briefly described, the main contributions of this work are: (1) harnessing the combined advantage of atlas- and CNN-based segmentations producing superior accuracies and test-retest precision when compared with other established atlas-based techniques (FSL, Freesurfer) and deep learning approaches (QuickNat, HippoDeep); (2) incorporating MRI data and corresponding hippocampus segmentations from patients with multiple brain disorders acquired with different MRI scanners and different field strengths (1.5 and 3.0 T) to improve the validity, robustness, and the general application of DeepHarp; and (3) designing an automatic method for automatic hippocampus segmentation following the ADNI-HarP standardization.

## 2. Materials and Methods

### 2.1. Datasets

The T1-weighted MRI datasets included in this study were acquired using various 3T and 1.5T MRI scanners and were collected from four sources: Sunnybrook Health Sciences Centre, Ontario, Canada (*n* = 50; datasets included healthy subjects and patients with various pathologies such as AD, AD with cerebral vascular disease, and MCI) [12], Alzheimer Disease Neuroimaging Initiative Harmonised Protocol database (ADNI-HarP; *n* = 130) [22], the Open Access Series of Imaging Studies (OASIS) Dementia Study (*n* = 14) [28], and a free online repository of healthy subjects and patients with epilepsy (*n* = 50) [11].

ADNI was launched in 2003 as a public-private partnership led by Principal Investigator, Michael W. Weiner, MD. The primary goal of ADNI has been to test whether serial MRI, positron emission tomography (PET), other biological markers, and clinical and neuropsychological assessments can be combined to measure the progression of mild cognitive impairment (MCI) and early Alzheimer’s disease (AD). For up-to-date information, see www.adni-info.org.

Finally, a freely available test-retest dataset of healthy subjects [29] was used for comparing the robustness of the proposed method with previously described hippocampus segmentation methods. From this dataset, 40 T1-weighted datasets from a single subject were used for this study, which were acquired in 20 sessions spanning 31 days using the ADNI-recommended protocol.

### 2.2. Pre-Processing

The image datasets were acquired on various scanners using distinct imaging parameters. Thus, the image resolution and signal intensities were not directly comparable between the datasets used to train the CNN. To overcome this limitation, the datasets were pre-processed by normalizing the intensity values to a mean of zero with a standard deviation of one and resampling to a 1 × 1 × 1 mm^3^ resolution with cubic interpolation.

### 2.3. ROI Identification

**Figure 1:**
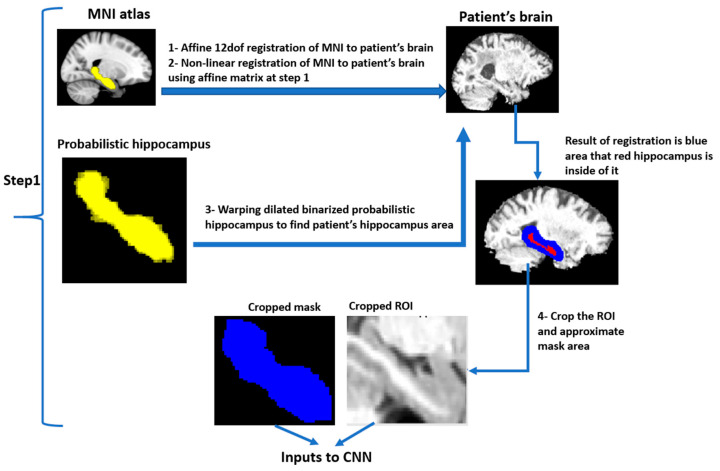
Atlas-based segmentation of the hippocampus involves the following sequential processes: (1) linear alignment of the Montreal Neurological Institute (MNI) brain atlas to the individual brain scans; (2) non-linear registration of the MNI atlas to the T1-weighted image; (3) warping the dilated binarized mask of the hippocampus to the patient space; and (4) cropping the region of interest.

**Figure 2:**
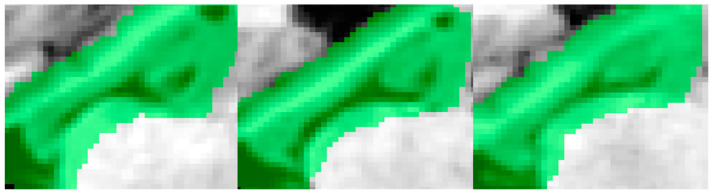
Three sagittal MRI bounding boxes and hippocampal ROIs (green), corresponding to three different patients. The ROI includes the hippocampus, and part of the white matter, Cerebro Spinal Fluid (CSF), and amygdala.

The pre-processing step of the proposed hippocampus segmentation method aims to detect a volume-of-interest (VOI) that contains the hippocampus within the 3D MRI datasets (see Figure 1 and Figure 2). A safety margin of two voxels in all three dimensions was used. This VOI margin helps to restrict the CNN training and testing to the medial temporal lobe rather than the whole image, which also improves class balance. Therefore, the Montreal Neurological Institute (MNI) brain atlas was first registered to the brain of each subject using an affine transformation followed by a non-linear registration step using cross-correlation as the similarity metric and spline interpolation implemented in the NiftyReg package [30]. The non-linear transformation was then applied to a probabilistic map of the hippocampus from the Harvard Oxford subcortical structural atlas [30]. The registered probabilistic map was binarized (every non-zero pixel was set to 1) and dilated by two voxels to assure that the final VOI included all parts of the hippocampus. The dilated hippocampus segmentation was then used to crop the MRI with the corresponding bounding box.

### 2.4. CNN Algorithm

**Figure 3:**
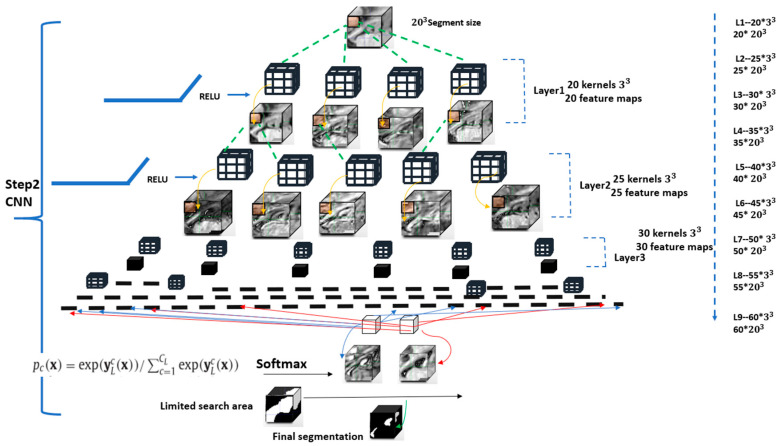
Structure of DeepHarp method: a pyramid structure with nine hidden layers.

The CNN architecture (Figure 3) described below aims to classify each voxel within the dilated hippocampus mask (green in Figure 2) as either background or foreground. All voxels outside the dilated hippocampal mask but within the bounding box are defined as background.

The fully-connected DeepMedic CNN architecture, originally designed for brain lesion segmentation and previously described in detail elsewhere [31], was optimized for hippocampus segmentation in this work. The DeepMedic framework is open source and publicly available on github.com. Despite its original development for brain lesion segmentation, it can serve as a general framework for easy creation and training of neural networks for the processing of medical images. DeepMedic employs a dual pathway architecture that enables the integration of local and larger contextual information, whereas one pathway uses the image at the original resolution while the second pathway uses a low-resolution version of the image. Since the larger contextual information is not needed due to the registration-based identification of a region-of-interest containing the hippocampus, DeepHarp for hippocampus segmentation only uses the high-resolution pathway. This high-resolution pathway is essentially a feedforward network that was configured to nine hidden layers using 3 × 3 × 3 kernels and a classification layer, which uses 1D kernels. Briefly described, the model used convolution layers followed by rectified linear unit (RELU) activation functions and a no max-pooling approach to preserve the image information [32]. Multiple configurations were used for fine tuning the base architecture of the CNN based on parameters previously described in literature [33]. More precisely, the number of hidden layers, learning rate, cost function, shape of network (pyramid) were tuned manually by using reference values from literature.

In the final setup, the number of feature maps started at 20 and increased by increments of five in each layer up to a maximum of 60 in the final layer. For the training stage, a batch size of 20 and 10 epochs were used to train the network by minimizing the cross-entropy loss function. Small 3 × 3 × 3 kernels were applied, which reduces the number of network parameters [31]. A funnel shape network was used because it has been shown to lead to superior performance [33] compared to networks with other shapes using the same number of parameters. The learning rate hyperparameter was initially set to 0.006 and steadily diminished until the validation accuracy plateaued. The L1 and L2 regularization terms were set to 0.000001 and 0.0001, respectively. Dropout was used to reduce the risk of model overfitting [31].

### 2.5. Training of DeepHarp

ADNI-HarP segmentations are specifically harmonized at the most posterior, anterior, and medial anatomical boundaries [22]. Hippocampus segmentations that were previously generated using non-ADNI-HarP protocols differed on average by more than one voxel in all orthogonal planes [22]. The hippocampus is a small structure with a large surface-area-to-volume ratio so that even a one voxel boundary error can introduce considerable volumetric differences.

The non-ADNI-HarP data from the Sunnybrook, OASIS, and the epileptic datasets used in this work were segmented using different manual labeling protocols, which vary based on local manual segmentation protocol. However, these manual labels encompassed most of the hippocampal anatomy included in the ADNI-HarP protocol. Thus, it was assumed that these datasets still provide useful information for training of the CNN-based segmentation model. The ADNI-HarP data that was used in this study was comprised of 130 MRI brain scans, of which 78 were used for training, together with all non-ADNI-HarP datasets (see Table 1). During training, the network parameters were adjusted based on validation set, which included 29 brain MRI scans from ADNI-HarP data not used in the training set. After training, the model was tested using 23 MRI brain scans from the ADNI-HarP data that were not used as part of the training or validation set. Thus, the test set of 23 ADNI-HarP brain MRI scans were never used for model training or optimization, and thus, represents an independent test set used to evaluate the performance of the model on unseen data.

**Figure 4:**
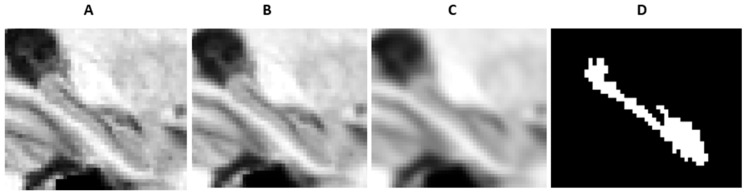
An augmented hippocampal ROI of the medial temporal lobe, using progressive Gaussian blurring, original ROI (**A**) (blurring with sigma 0.5 (**B**), with sigma 1.0 (**C**)) and corresponding ground truth segmentation (**D**).

Thus, DeepHarp was trained using anatomical segmentation patterns from a combination of the local-protocols and the ADNI-HarP protocol. To improve the generalizability of the CNN model, all training data were augmented by Gaussian blurring using standard deviations of 0.5 and 1.0 (Figure 4).

### 2.6. Evaluation of the DeepHarp Model

For validation purposes, the hippocampus segmentation results obtained by the DeepHarp model were compared to the segmentation results generated using FSL [17] and Freesurfer [15]. We additionally compared our results to the recently reported CNN-based segmentation methods QuickNat [14] and HippoDeep [13]. The Dice similarity metric was used as a quantitative overlap measurement for segmentation quality assessment. Dice values close to 1.0 indicate a perfect consensus with the de facto ground-truth labels (i.e., expert manual segmentations) while values close to 0 indicate no consensus. Twenty-three test datasets (20% of the ADNI-HarP data that were not used for training or validation) were used for accuracy testing.

The precision of the proposed segmentation method was evaluated using two independent MRI scans of the same subject available for each scan session (20 sessions in total). For a quantitative evaluation, the second MRI of each session was registered to the first image acquisition of the same session, using a rigid transformation with a normalized cross-correlation cost function and linear interpolation. After this step, the previously described preprocessing steps and DeepHarP method were applied to each of the co-registered images for hippocampus segmentation. The two segmentations from each session were compared using the Dice coefficient, and the average Dice coefficient was computed across all sessions. As all scan pairs were acquired from the same subject within one-month, minimal volume changes were expected over this interval, and the Dice similarity metric was hypothesized to be close to 1.0. We additionally performed hippocampus segmentation on these 20 scan pairs using the FSL [17], Freesurfer [15], QuickNat [14], and HippoDeep [13] methods for comparison with the proposed DeepHarP method.

### 2.7. Implementation Details

The training of the CNN model was performed using a NVIDIA GTX 1060 GPU and the CUDA 9 toolkit and cuDNN 7.5 with TensorFlow 1.12.1. All pre-processing steps were performed using the visualization toolkit (VTK) and python 2.7. All registrations were performed using the NiftyReg package [30]. Brain extraction and bias field correction were done using the brain extraction tool (BET), part of the FSL package. All statistical analyses were performed using open-source R and RStudio software packages. The trained model, pre-processing scripts and instructions for using tool are available on https://github.com/snobakht/DeepHarP_Hippocampus/tree/main/DeepHarP.

## 3. Results

The quantitative evaluation of the segmentations generated showed that the proposed DeepHarp model achieved an average Dice score of 0.88 ± 0.02 using the 23 test datasets (46 hippocampi segmented from ADNI-Harp data). No significant difference was observed regarding the Dice similarity metrics of the left and right hippocampi.

**Figure 5:**
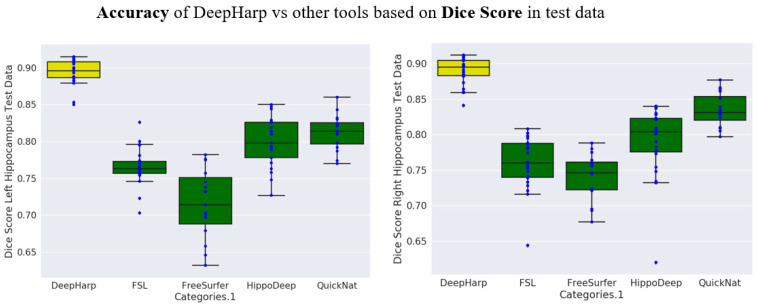
Segmentation accuracy of DeepHarp and the other four tools on the test set.

**Figure 6:**
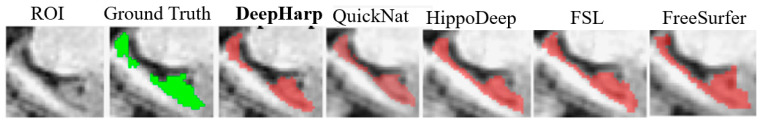
Comparison of segmentations for different tools for a selected patient and slice. It can be seen that Freesurfer and FSL show more false positives than other tools at the head of hippocampus. Freesurfer also has more false negatives at the tail than other tools.

The quantitative comparison to the four other established hippocampus segmentation techniques (i.e., FSL, QuickNat, Freesurfer, and HippoDeep) showed that DeepHarp achieved significantly higher Dice scores (Wilcoxon rank-sum test, *p* < 0.001) indicating a high level of accuracy (Figure 5). Figure 6 shows a selected segmentation for a subject generated by the proposed method and the other four methods. Overall, the three deep learning methods that were evaluated (i.e., DeepHarp, QuickNat, and HippoDeep) generated segmentations that were more similar to the ground truth segmentations compared to Freesurfer and FSL, which is in line with the quantitative results. Table 2 shows the comparisons and *p*-values.

### Precision

**Figure 7:**
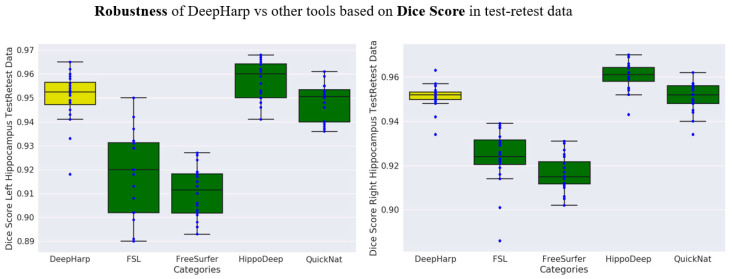
Comparison of DeepHarp robustness (based on Dice score) with common tools and state-of-the-art methods for hippocampus segmentation.

**Figure 8:**
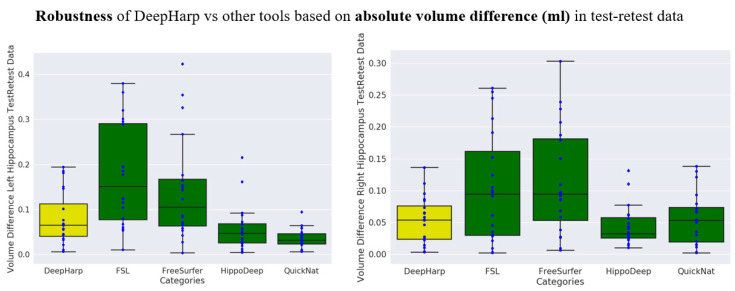
Comparison of DeepHarp robustness (based on absolute volume difference) with common tools and state-of-the-art methods for hippocampus segmentation.

The test-retest dataset was used for comparing the robustness of the proposed DeepHarp method with FSL, QuickNat, Freesurfer, and HippoDeep. The test-retest data was acquired using the same scanner and acquisition parameters and should present only minor volumetric differences since the two datasets compared for each day were acquired within the same imaging session, but with repositioning of the participant between the two scans. Figure 7 shows the performance of the five segmentation techniques by means of the Dice score. The quantitative evaluation revealed an average Dice score of 0.95 ± 0.01, with no significant difference between right and left hippocampus segmentations for the proposed DeepHarp method. Figure 8 shows the performance of the five techniques based on absolute volume difference in milliliter. DeepHarp measured an average difference of 0.08 ± 0.057 and 0.05 ± 0.036 milliliter between pairs of test-retest data for the left hippocampus and right hippocampus, respectively.

The statistical evaluation on Dice scores and absolute volume difference revealed that DeepHarp is significantly more robust than FSL for the left hippocampus and Freesurfer for the right hippocampus segmentation (Wilcoxon rank-sum test, *p* < 0.003 and *p*< 0.01). HippoDeep is significantly more robust than DeepHarp (Wilcoxon rank-sum test, largest *p* < 0.03) based on Dice scores, while no statistically significant difference was found between HippoDeep and DeepHarp (Wilcoxon rank-sum test, *p* < 0.1) based on volume differences. DeepHarp and QuickNat have no significant difference based on Dice scores, while QuickNat is statistically more robust than DeepHarp on left hippocampus measurements, but not for the right hippocampus. Overall, DeepHarp, QuickNat, and HippoDeep are comparable in robustness based on the two quantitative metrics, Dice scores and absolute volume differences. Table 3 shows the detailed data and *p*-values.

The initial development and evaluation of DeepHarp was performed using full non-cropped images without atlas segmentation. This took more than three days to complete for every round of training. Utilization of an atlas-based approach significantly improved the training time and results. Therefore, every training trial on the full set of data took approximately 6h to complete. This relatively short training time allowed for many rounds of trial and errors to identify the best parameters and hyperparameters. Excluding the atlas-based segmentation, DeepHarp segments the hippocampus in an MRI dataset in about 6 s. Adding the atlas-based segmentation can increase this time to 10 min, depending on whether a GPU is available.

## 4. Discussion

This study shows that combining a traditional atlas-based segmentation method with an optimized CNN architecture leads to improved segmentation accuracies compared to CNN- and atlas-based segmentation algorithms alone. Providing prior knowledge to the CNN, which was derived by an atlas-based method, limits the search area, and increases the foreground to background ratio, which likely contributed to the high accuracy of DeepHarp. The DeepHarp method achieved a mean Dice similarity score of 0.88, which was significantly better than four other established hippocampus segmentation methods used for comparison. At the same time, the proposed method also achieved a high test-retest precision (mean Dice score: 0.95). Overall, DeepHarp achieves higher accuracy and test-retest precision than commonly used segmentation tools such as FSL and Freesurfer, and achieves more accurate results compared to other deep learning methods [13,14].

Automation of the ADNI-HarP protocol is necessary to increase efficiency, accuracy, and standardization of hippocampus segmentations within larger studies. Therefore, a major contribution of this work is that the proposed DeepHarp technique achieves highly accurate and precise segmentation results following the ADNI-HarP standard. The proposed model demonstrated high accuracy and precision across different scanners, image resolutions, and pathologies. Due to the limited number of manually segmented datasets from the original ADNI-HarP validation sample, we utilized datasets that were manually segmented by medical experts, using validated protocols with inclusive anatomical definitions. To assure that the final model segments the hippocampus according to the ADNI-HarP guidelines, the trained CNN model was additionally fine-tuned using an independent validation sample selected from the ADNI-HarP dataset, which was then tested using another independent ADNI-HarP sample.

Another major advantage of the proposed method is its simplicity, as it only requires an atlas registration and subsequent application of the trained CNN. Although training of a CNN can be time-consuming, the application of a trained network is usually quite fast using modern hardware especially if applied to a small ROI.

Excluding the atlas-based segmentation, DeepHarp segments the hippocampus in an MRI dataset in about 6 s. The efficient DeepMedic architecture as well as cropping images using the atlas-based segmentation are key components for fast inference time of DeepHarp. However, the atlas registration can take up to 10 min depending on the implementation and computational resources available. The other two deep learning methods used in this work for comparison (QuickNat and HippoDeep) require less than a minute for a single dataset while FreeSurfer and FSL are considerably slower. However, it needs to be highlighted that there is no clinical decision that requires fast hippocampus segmentation from MRI. What matters most is a reliable, robust, and accurate segmentation for use as an image-based biomarker in clinical decision making and clinical studies.

From a technical perspective, the main contribution of this work is the development and evaluation of an advanced method for hippocampus segmentation from T1-weigthed MRI datasets, according to the ADNI-HarP protocol by combining an atlas-based and CNN-based segmentation methods. These two techniques are the two most used general approaches for hippocampus segmentation [13,14,24,25,26,27]. However, to the best of our knowledge, DeepHarp is the first method to actually combine these two general approaches.

However, there are still many interesting avenues to further improve the accuracy of DeepHarp. For example, a recent study by Dill et al. [26] showed that using meta data such as patient’s age, sex, clinical status can improve the Dice coefficient of an atlas-based hippocampus segmentation by 5%. Thus, it might be interesting to also include such meta data in DeepHarp. Another study by Zhu et al. [25] proposed metric learning for improved atlas-based hippocampus segmentation. The authors of that study developed a novel label fusion technique by using a distance metric where similar image patches are kept close together and non-similar patches are grouped far from each other. Their method dynamically determines a distance metric rather than using a predefined distance metric as done in previous methods. When that method was applied to ADNI data, it achieved a statistically significant improvement in hippocampus segmentation accuracy. For this reason, it might be beneficial to use this advanced atlas-based segmentation method instead of the rather simple atlas-based segmentation method used in DeepHarp. However, due to the subsequent application of a CNN in DeepHarp, the actual benefit might be insignificant. A study by Liu et al. [27] proposed a multi-modal convolutional network to identify hippocampus features using a multi-task CNN and then used a dense network to use these features for a classification task (AD vs. normal control subjects). Their model achieved 87% accuracy for classifying AD, MCI, and normal control subjects. Thus, it might be interesting to also implement a subsequent disease classification in DeepHarp. Another study by Sun et al. [24] describes a dual convolutional neural network for hippocampus segmentation, which uses a unique loss function in a v-net structure [34] and achieves 90% accuracy, which could also prove beneficial for DeepHarp.

DeepHarp is based on the DeepMedic architecture [31] that has been used for various segmentations tasks and generally performs well for many biomedical image segmentations problems. In this study, baseline comparison to four other established models described in literature were performed, showing that the proposed model is more accurate and as reliable compared to previously described methods. By now, there are hundreds of different CNN networks described in the literature (e.g., U-Nets [35], MASK-RCNN [36], VGG16 [37], VGG19 [38], ResNet [39], and AlexNet [40]). It is likely that at least one of these models will perform better than the proposed model based on DeepMedic architecture, and it may only be because of overfitting or random chance. However, testing every single CNN base model for hippocampus segmentation is virtually impossible and outside the scope of this paper. The primary goal of this work was to show the effectiveness of combining atlas-based segmentation with a deep learning model for hippocampus segmentation without the requirement of a massive amount of data. Within this context, it is likely that another CNN method would achieve similar results compared to the DeepMedic architecture when combined with an atlas-based segmentation approach. Thus, this work should be considered a proof-of-principle, showing the effectiveness of combined atlas- and CNN-based segmentation models that are potentially translatable to many other segmentation problems.

In our study, the selection of data used for training DeepHarp was very diverse, as demonstrated by the inclusion of MRI scans from patients with multiple brain disorders (dementia, epilepsy and normal controls) and dementia subtypes (MCI, AD, VCI) [11,12,21,22,23,28]. Although previous studies describing CNN-based hippocampus segmentation methods have used diverse datasets, the data selection used for training of DeepHarp is much more diverse due to its acquisition at multiple clinical sites using MRI scanners from different vendors with varying field strengths. The use of such datasets and the combined application of atlas- and CNN-based methods are likely the key reasons that make DeepHarp very accurate and robust. This is an important feature to consider since a major drawback of using deep learning models is their inability to perform well if the distribution of test images is different than training sets [41]. We compared our method quantitatively with four published segmentation methods whereas the quantitative results show that the proposed method outperformed four other published segmentation methods with respect to the accuracy and achieved comparable robustness.

There are several limitations to our proposed method that should be addressed in future work. First, the use of non-linear registration in the pre-processing stages can be time consuming. Future work should investigate if an affine registration might be suitable enough for this purpose. Second, the CNN generates segmentations using T1-weighted data only. Whether the proposed method can be applied/re-trained to other MRI sequences or multi-modal MRI datasets requires further assessment and is beyond the scope of this study. Although our method yielded high accuracy, the reliability of the DeepHarp was significantly lower than the HippoDeep method. This is likely due to the higher number of training sets used in HippoDeep (>20 k). Including auxiliary data (using automated tools) for post training of DeepHarp may improve robustness but likely at the price of reduced accuracy. In this case, robustness is a measure of segmentation consistency across different time points. In other words, for this specific analysis, we mostly investigated if the methods make the same mistakes for the same patients or if the segmentation errors occur at different locations when segmenting the same structure in the same patient in images acquired at multiple time points. This analysis shows that all methods make similar mistakes across different imaging time points, while DeepHarp achieves the highest segmentation accuracy when comparing the different methods. Thus, it is important to consider the accuracy levels found when comparing the robustness between models. In other words, the results suggest that DeepHarp is precise and accurate.

From a clinical perspective, DeepHarp improved hippocampus segmentation accuracy is important as it could help to establish an image-based biomarker for early detection of Alzheimer’s disease that can be used in prospective clinical testing of the effects of disease modifying treatments on rates of hippocampus atrophy as a secondary outcome measure.

## 5. Conclusions

Our findings suggest that the DeepHarp can automatically segment the hippocampus from T1-weighted MRI datasets according to the ADNI-HarP protocol with higher accuracy than four other state-of-the-art methods, while also achieving high precision.

## Figures and Tables

**Table 1:** Training, validation, testing, and test-retest data count.

Datasets	ADNI-HarP Segmentation	Total Sample Size	Training Data	Validation Data	Testing Data	Test-Retest Data
SunnyBrook	no	50	50	0	0	0
OASIS	no	14	14	0	0	0
ADNI-HarP	yes	130	78	29	23	0
Healthy and patients with epilepsy	no	50	50	0	0	0
Test-retest (single subject)	no	40	0	0	0	40
Total	-	284	192	29	23	40

**Table 2:** Accuracy and precision of the DeepHarp method compared to other methods: the metric is the Dice coefficient. The bold numbers are showing highest Dice scores. DeepHarp has highest Dice score in accuracy and HippoDeep has highest Dice score in robustness.

	Test Set/Dice Coefficient				Test-Retest Set/Dice Coefficient			
	Left Hippocampus Mean ± SD	Right Hippocampus Mean ± SD	*p*-Values vs. DeepHarp Left Hippocampus	*p*-Values vs. DeepHarp Right Hippocampus	Left Hippocampus Mean ± SD	Right Hippocampus Mean ± SD	*p*-Values vs. DeepHarp Left Hippocampus	*p*-Values vs. DeepHarp Right Hippocampus
DeepHarp	**0.893 ± 0.017**	**0.889 ± 0.190**	N/A	N/A	0.95 ± 0.01	0.951 ± 0.005	N/A	N/A
HippoDeep	0.799 ± 0.033	0.79 ± 0.049	<0.001	<0.001	**0.957 ± 0.008**	**0.96 ± 0.006**	0.029	<0.001
QuickNat	0.812 ± 0.024	0.834 ± 0.023	<0.001	<0.001	0.948 ± 0.007	0.951 ± 0.006	0.278	0.817
FSL	0.765 ± 0.025	0.759 ± 0.037	<0.001	<0.001	0.918 ± 0.017	0.923 ± 0.012	<0.001	<0.001
FreeSurfer	0.715 ± 0.047	0.741 ± 0.033	<0.001	<0.001	0.91 ± 0.01	0.916 ± 0.008	<0.001	<0.001

**Table 3:** Comparison of DeepHarp with other tools regarding robustness. The bold numbers in second and third columns show DeepHarp results. The bold numbers in sixth and seventh columns show highest robustness Dice scores belonging to HippoDeep.

	Test-Retest Set/Absolute Volume Differences in Milliliter				Test-Retest Set/Dice Scores			
	Left Hippocampus Mean Volume Difference ± SD	Right Hippocampus Mean Volume Difference ± SD	*p*-Values vs. DeepHarp Left Hippocampus	*p*-Values vs. DeepHarp Right Hippocampus	Left Hippocampus Mean Dice ± SD	Right Hippocampus Mean Dice ± SD	*p*-Values vs. DeepHarp Left Hippocampus	*p*-Values vs. DeepHarp Right Hippocampus
DeepHarp	**0.080 ± 0.057**	**0.052 ± 0.036**	N/A	N/A	0.95 ± 0.01	0.951 ± 0.005	N/A	N/A
HippoDeep	0.056 ± 0.050	0.044 ± 0.030	<0.163	<0.622	**0.957 ± 0.008**	**0.96 ± 0.006**	0.029	<0.001
QuickNat	0.035 ± 0.021	0.055 ± 0.040	<0.004	<0.881	0.948 ± 0.007	0.951 ± 0.006	0.278	0.817
FSL	0.174 ± 0.11	0.103 ± 0.085	<0.003	<0.101	0.918 ± 0.017	0.923 ± 0.012	<0.001	<0.001
FreeSurfer	0.141 ± 0.113	0.114 ± 0.082	<0.078	<0.010	0.91 ± 0.01	0.916 ± 0.008	<0.001	<0.001

## Data Availability

Data sharing not applicable.

## References

[1] Knopman D.S. (2007). Dementia and cerebrovascular disease. Res. Pract. Alzheimers Dis..

[2] WHO Dementia: A Public Health Priority. https://www.who.int/mental_health/publications/dementia_report_2012/en/.

[3] Lista S., Garaci F.G., Ewers M., Teipel S., Zetterberg H., Blennow K., Hampel H. (2014). CSF Aβ1-42 combined with neuroimaging biomarkers in the early detection, diagnosis and prediction of Alzheimer’s disease. Alzheimer’s Dement..

[4] Tondelli M., Wilcock G.K., Nichelli P., De Jager C.A., Jenkinson M., Zamboni G. (2012). Structural MRI changes detectable up to ten years before clinical Alzheimer’s disease. Neurobiol. Aging.

[5] Jack C.R., Wiste H.J., Weigand S.D., Knopman D.S., Mielke M.M., Vemuri P., Lowe V.J., Senjem M.L., Gunter J.L., Reyes A.D. (2015). Different definitions of neurodegeneration produce similar amyloid/neurodegeneration biomarker group findings. Brain.

[6] Yu P., Sun J., Wolz R., Stephenson D., Brewer J., Fox N.C., Cole P.E., Jack C.R., Hill D.L.G., Schwarz A.J. (2012). Operationalizing hippocampal volume as an enrichment biomarker for amnestic MCI trials: Effect of algorithm, test-retest variability and cut-point on trial cost, duration and sample size. Neurobiol. Aging.

[7] Wolz R., Schwarz A.J., Gray K.R., Yu P., Hill D.L., Initiative A.D.N. (2016). Enrichment of clinical trials in MCI due to AD using markers of amyloid and neurodegeneration. Neurology.

[8] Den Heijer T., Geerlings M.I., Hoebeek F.E., Hofman A., Koudstaal P.J., Breteler M.M.B. (2006). Use of hippocampal and amygdalar volumes on magnetic resonance imaging to predict dementia in cognitively intact elderly people. Arch. Gen. Psychiatry.

[9] Martin S.B., Smith C.D., Collins H.R., Schmitt F.A., Gold B.T. (2010). Evidence that volume of anterior medial temporal lobe is reduced in seniors destined for mild cognitive impairment. Neurobiol. Aging.

[10] Heijer T.D., Van Der Lijn F., Koudstaal P.J., Hofman A., Van Der Lugt A., Krestin G.P., Niessen W.J., Breteler M.M.B. (2010). A 10-year follow-up of hippocampal volume on magnetic resonance imaging in early dementia and cognitive decline. Brain.

[11] Jafari-Khouzani K., Elisevich K.V., Patel S., Soltanian-Zadeh H. (2011). Dataset of magnetic resonance images of nonepileptic subjects and temporal lobe epilepsy patients for validation of hippocampal segmentation techniques. Neuroinformatics.

[12] Nestor S.M., Gibson E., Gao F.Q., Kiss A., Black S.E. (2013). A direct morphometric comparison of five labeling protocols for multi-atlas driven automatic segmentation of the hippocampus in Alzheimer’s disease. Neuroimage.

[13] Thyreau B., Sato K., Fukuda H., Taki Y. (2018). Segmentation of the hippocampus by transferring algorithmic knowledge for large cohort processing. Med. Image Anal..

[14] Roy A.G., Conjeti S., Navab N., Wachinger C. (2018). QuickNAT: Segmenting MRI Neuroanatomy in 20 seconds. arXiv.

[15] Pipitone J., Park M.T.M., Winterburn J., Lett T.A., Lerch J.P., Pruessner J.C., Lepage M., Voineskos A.N., Chakravarty M.M. (2014). Multi-atlas segmentation of the whole hippocampus and subfields using multiple automatically generated templates. Neuroimage.

[16] Patenaude B., Smith S.M., Kennedy D.N., Jenkinson M. (2011). A Bayesian model of shape and appearance for subcortical brain segmentation. Neuroimage.

[17] Géron A. (2017). Hands-On Machine Learning with Scikit-Learn.

[18] Papandreou G., Chen L.-C., Murphy K., Yuille A.L. (2015). Weakly- and Semi-Supervised Learning of a DCNN for Semantic Image Segmentation. arXiv.

[19] Fischl B. (2013). FreeSurfer. Neuroimage.

[20] Dolz J., Desrosiers C., Ben Ayed I. (2018). 3D fully convolutional networks for subcortical segmentation in MRI: A large-scale study. Neuroimage.

[21] Duchesne S., Valdivia F., Robitaille N., Mouiha A., Bocchetta M., Apostolova L.G., Ganzola R., Preboske G., Wolf D., Boccardi M. (2015). Manual segmentation qualification platform for the EADC-ADNI harmonized protocol for hippocampal segmentation project. Alzheimer’s Dement..

[22] Duchesne S., Valdivia F., Robitaille N., Valdivia A., Apostolova L., Bocchetta M., Ganzola R., Preboske G., Wolf D., Boccardi M. (2013). Manual segmentation certification platform for the EADC-ADNI harmonized protocol for the hippocampal volumetry project. Alzheimer’s Dement..

[23] Sun J., Yan S., Song C., Han B. (2020). Dual-functional neural network for bilateral hippocampi segmentation and diagnosis of Alzheimer’s disease. Int. J. Comput. Assist. Radiol. Surg..

[24] Zhu H., Tang Z., Cheng H., Wu Y., Fan Y. (2019). Multi-atlas label fusion with random local binary pattern features: Application to hippocampus segmentation. Sci. Rep..

[25] Dill V., Klein P.C., Franco A.R., Pinho M.S. (2018). Atlas selection for hippocampus segmentation: Relevance evaluation of three meta-information parameters. Comput. Biol. Med..

[26] Liu M., Li F., Yan H., Wang K., Ma Y., Shen L., Xu M. (2020). A multi-model deep convolutional neural network for automatic hippocampus segmentation and classification in Alzheimer’s disease. Neuroimage.

[27] Ataloglou D., Dimou A., Zarpalas D., Daras P. (2019). Fast and Precise Hippocampus Segmentation through Deep Convolutional Neural Network Ensembles and Transfer Learning. Neuroinformatics.

[28] Pamela J., Ls T., John C., Andrei G., Marcus E. (2019). OASIS-3: Longitudinal Neuroimaging, Clinical, and Cognitive Dataset for Normal Aging and Alzheimer Disease. MedRxiv.

[29] Maclaren J., Han Z., Vos S.B., Fischbein N., Bammer R. (2014). Reliability of brain volume measurements: A test-retest dataset. Sci. Data.

[30] Modat M., Cash D.M., Winston G.P., Duncan J.S. (2014). Global image registration using a symmetric block-matching approach. J. Med. Imaging.

[31] Kamnitsas K., Ledig C., Newcombe V.F., Simpson J.P., Kane A.D., Menon D.K., Rueckert D., Glocker B. (2017). Efficient multi-scale 3D CNN with fully connected CRF for accurate brain lesion segmentation. Med. Image Anal..

[32] Long J., Shelhamer E., Darrell T. Fully convolutional networks for semantic segmentation. Proceedings of the IEEE Conference on Computer Vision and Pattern Recognition.

[33] Bengio Y. (2012). Practical recommendations for gradient-based training of deep architectures. Lect. Notes Comput. Sci..

[34] Milletari F., Navab N., Ahmadi S.A. V-Net: Fully convolutional neural networks for volumetric medical image segmentation. Proceedings of the 2016 fourth international conference on 3D vision (3DV).

[35] Ronneberger O., Fischer P., Brox T. U-Net: Convolutional Networks for Biomedical Image Segmentation. Proceedings of the International Conference on Medical Image Computing and Computer-Assisted Intervention.

[36] He K., Gkioxari G., Dollár P., Girshick R. (2017). Mask, r-cnn. arXiv.

[37] van Opbroek A., Achterberg H.C., Vernooij M.W., Arfan Ikram M., de Bruijne M. (2018). Transfer learning by feature-space transformation: A method for Hippocampus segmentation across scanners. NeuroImage Clin..

[38] Simonyan K., Zisserman A. Very deep convolutional networks for large-scale image recognition. Proceedings of the 3rd International Conference on Learning Representations ICLR 2015.

[39] Mateen M., Wen J., Nasrullah Song S., Huang Z. (2019). Fundus image classification using VGG-19 architecture with PCA and SVD. Symmetry.

[40] He K., Zhang X., Ren S., Sun J. Deep residual learning for image recognition. Proceedings of the IEEE Conference on Computer Vision and Pattern Reco4gnition 2016.

[41] Alex K., Sutskever I. (2012). GEH. Advances in Neural Information Processing Systems 25 (NIPS 2012).

